# The relationship between alexithymia and Nyght Eating Syndrome

**DOI:** 10.1192/j.eurpsy.2023.906

**Published:** 2023-07-19

**Authors:** F. Micanti, E. Amoroso, M. Vannini, C. Ricci, G. Spennato, M. D’Ambrosio, M. Billeci, H. Lamberti

**Affiliations:** Psychiatry and Psychology, “Federico II” University Medical School, Naples, Italy

## Abstract

**Introduction:**

NES is characterized by daytime anorexia, sleep difficulties with nocturnal food intake, resulting in obesity (*Stunkard et al.* Am J of Med. 1955; 19 78-86). Alexithymia refers to the impairment in recognizing and describing feelings. The impairment in distinguishing emotions from body sensations may lead patients to confuse emotional arousal with physical hunger (*Sifneos et al.* Mod. trends psychosom. med. 1976; 3 430-439). This mechanism could lead to nocturnal food intake. Alexithymia was firstly described in BED and was related to BED severity.

**Objectives:**

To our knowledge no studies have investigated the relationship between alexithymia and NES. The aim of the present study was to assess alexithymia in patients with NES, to improve surgical and nutritional outcomes.

**Methods:**

110 patients with clinical diagnosis of NES admitted to the Eating Disorder Unit, between 2013 and 2022 underwent psychiatric assessment for bariatric surgery. Clinical assessment consisted of clinical interview and the following psychometric rating scales: 20-item Toronto Alexithymia Scale; Eating Disorder Inventory 2, specifically the Interoceptive Awareness subscale; Barratt Impulsiveness Scale; Binge Eating Scale.

**Results:**

The mean BES score was 24.14(SD 8.23), computed on 107 patients, of which 16 (14.5%) had no or minimal binge eating problems and 91 (82.7%) had moderate-severe binge eating problems. The mean TAS total score was 55.11(12.92), computed on 103 patients. 42 patients had a TAS-20 total score ≤50 and were categorized as non-alexithymic, and 61 had a TAS-20 total score >50 and were categorized as alexithymic. Simple linear regression was used to test if TAS-20 total score significantly predicted EDI-IA in the whole sample (97 patients). The overall regression was statistically significant (R^2^=0.27, F(1,96)=35.46, p< .001) and TAS total score significantly predicted EDI-IA score (β=0.519, p<.001). In the alexithymic group, the regression was statistically significant (R^2^=0.305, F(1,57)=25.07, p< .001) and TAS total score significantly predicted EDI-IA score (β=0.553, p<.001).

**Table:** 

	**Whole sample**	**Alexithymic**	**Non-alexithymic**	***p***
Female (%)	79(71.8) N=110	48(64.9) N=62	26(35.1) N=42	.087
Age mean(SD)	36.41(12.6) N=110	34.65(12.7) N=62	38.48(11.31) N=42	.11
BMI mean(SD)	44.05(7.61) N=106	43.97(7.9) N=60	43.82(7.38) N=40	.92
TAS mean(SD)	55.11(12.92) N=103	64.05(7.06) N=61	42.12(7.13) N=42	<.001*
BIS mean(SD)	67.58(10.35) N=98	70.07(9.93) N=59	63.26 (9.85) N=35	.002*
BES mean(SD)	24.14(8.23) N=107	25.7(6.89) N=62	21(9.25) N=40	.004*
EDI-IA mean(SD)	8.95(6.44) N=103	10.82(7) N=60	6.05(4.44) N=39	.000*

* *significant difference between alexithymic and non-alexithymic groups according to independent sample t-test.*

**Image:**

**Figure:**
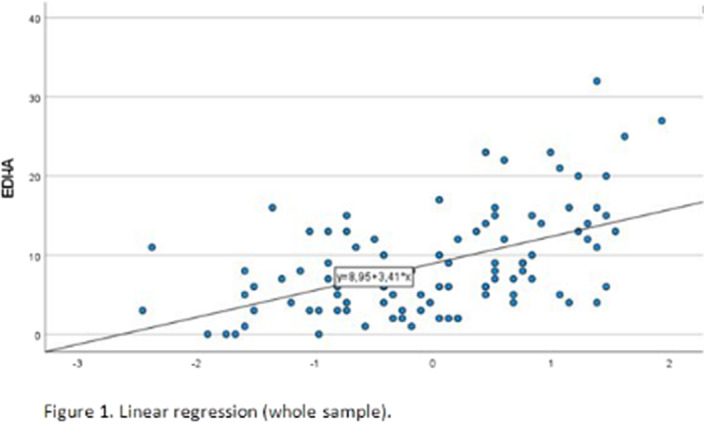

**Image 2:**

**Figure:**
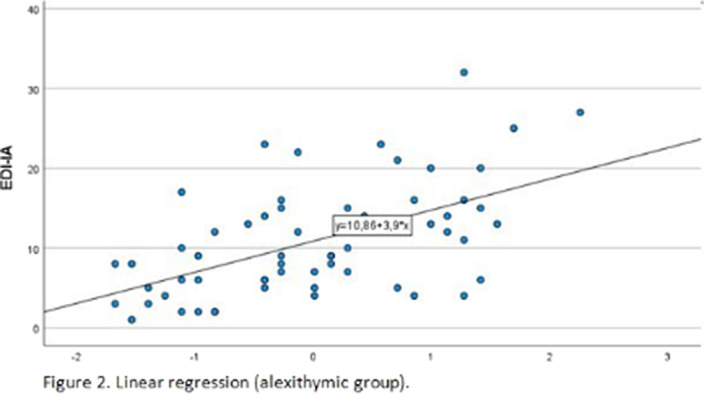

**Conclusions:**

In patients with NES, alexithymia significantly predicts poor interoceptive awareness, thus explaining excessive nocturnal food intake.

**Disclosure of Interest:**

None Declared

